# RETRACTION: Facial Emotion Recognition Using a Novel Fusion of Convolutional Neural Network and Local Binary Pattern in Crime Investigation

**DOI:** 10.1155/cone/9863126

**Published:** 2026-06-26

**Authors:** Computational Intelligence and Neuroscience

RETRACTION: D. Zhu, Y. Fu, X. Zhao, X. Wang, and H. Yi, “Facial Emotion Recognition Using a Novel Fusion of Convolutional Neural Network and Local Binary Pattern in Crime Investigation,” *Computational Intelligence and Neuroscience*, no. 2022 (2022), https://doi.org/10.1155/2022/2249417.

The above article, published online on 22 September 2022 in Wiley Online Library (https://wileyonlinelibrary.com), has been retracted by John Wiley & Sons Ltd.

This article has been retracted following an investigation undertaken by the publisher. This investigation has uncovered evidence of one or more of the following indicators of systematic manipulation of the publication process:

1. Discrepancies in scope
2. Discrepancies in the description of the research reported
3. Discrepancies between the availability of data and the research described
4. Inappropriate citations
5. Incoherent, meaningless and/or irrelevant content included in the article
6. Manipulated or compromised peer review

The presence of these indicators undermines our confidence in the integrity of the article’s content, and we cannot, therefore, vouch for its reliability. Please note that this notice is intended solely to alert readers that the content of this article is unreliable. We have not investigated whether authors were aware of or involved in the systematic manipulation of the publication process.

Xin Wang disagrees with this retraction. The other authors were informed of the retraction but did not provide a response.

